# The rexinoid V-125 reduces tumor growth in preclinical models of breast and lung cancer

**DOI:** 10.1038/s41598-021-04415-0

**Published:** 2022-01-07

**Authors:** Lyndsey A. Reich, Jessica A. Moerland, Ana S. Leal, Di Zhang, Sarah Carapellucci, Beth Lockwood, Peter W. Jurutka, Pamela A. Marshall, Carl E. Wagner, Karen T. Liby

**Affiliations:** 1 Department of Pharmacology and Toxicology, Michigan State University College of Osteopathic Medicine, B430 Life Science Building, 1355 Bogue Street, East Lansing, MI 48824 USA; 2 School of Mathematical and Natural Sciences, New College of Interdisciplinary Arts and Sciences, Arizona State University, Glendale, AZ USA

**Keywords:** Cancer, Drug discovery

## Abstract

Rexinoids are ligands which activate retinoid X receptors (RXRs), regulating transcription of genes involved in cancer-relevant processes. Rexinoids have anti-neoplastic activity in multiple preclinical studies. Bexarotene, used to treat cutaneous T cell lymphoma, is the only FDA-approved rexinoid. Bexarotene has also been evaluated in clinical trials for lung and metastatic breast cancer, wherein subsets of patients responded despite advanced disease. By modifying structures of known rexinoids, we can improve potency and toxicity. We previously screened a series of novel rexinoids and selected V-125 as the lead based on performance in optimized in vitro assays. To validate our screening paradigm, we tested V-125 in clinically relevant mouse models of breast and lung cancer. V-125 significantly (p < 0.001) increased time to tumor development in the MMTV-Neu breast cancer model. Treatment of established mammary tumors with V-125 significantly (p < 0.05) increased overall survival. In the A/J lung cancer model, V-125 significantly (p < 0.01) decreased number, size, and burden of lung tumors. Although bexarotene elevated triglycerides and cholesterol in these models, V-125 demonstrated an improved safety profile. These studies provide evidence that our screening paradigm predicts novel rexinoid efficacy and suggest that V-125 could be developed into a new cancer therapeutic.

## Introduction

The landscape of cancer therapy has shifted significantly in recent years from standard of care cytotoxic chemotherapy alone to targeted therapies and immunotherapy^1^. However, the need for additional therapeutics still exists, particularly for patients with late-stage disease, aggressive molecular subtypes or who have failed existing treatments. While specific monoclonal antibodies (trastuzumab) or small molecule inhibitors (lapatinib, neratinib) of HER2 (human epidermal growth factor receptor 2) confer a survival benefit to patients with HER2 + breast cancer, subsets of patients do not benefit from these drugs, and others acquire resistance via multiple mechanisms^2^. Patients with non-small cell lung cancer (NSCLC) also acquire resistance to chemotherapy^3^ or to targeted therapies^4^, contributing to disease progression.

Rexinoids are synthetic agonists which selectively bind to Retinoid X Receptors (RXR). Upon activation, RXR acts as a transcription factor for genes involved in several cancer-related biological processes, such as inflammation, proliferation, and cell survival^5^. Rexinoids are effective in multiple preclinical models of cancer and have been tested as therapeutic options for neurodegenerative and autoimmune diseases^6^. The rexinoid bexarotene was FDA approved in 1999 for the treatment of cutaneous T cell lymphoma (CTCL)^7^. Bexarotene has also been evaluated in clinical trials for both metastatic breast cancer^8^ and NSCLC^9^, and subsets of patients derived significant clinical benefit despite late-stage, aggressive disease. Newly synthesized rexinoids have improved anti-tumor efficacy and reduced toxicity in comparison to bexarotene^10,11^, which has revitalized interest in developing and testing rexinoids for cancer treatment.

The first studies of bexarotene in animal models of breast cancer were in the NMU rat model of mammary carcinogenesis, wherein the carcinogen *N*-nitro-*N*-methylurea (NMU) initiates the formation of estrogen receptor positive (ER +) mammary tumors^12^. In these studies, bexarotene reduced tumor burden by 90% and was well-tolerated^13^. While retinoids are active in this model^14,15^, they cause headaches and mucocutaneous toxicity in patients as a result of binding to the retinoic acid receptor (RAR)^16^. These side effects are not observed with the rexinoids that do not bind to RAR^17^. When the anti-tumor efficacy of bexarotene was compared directly to that of the selective estrogen receptor modulator (SERM) tamoxifen, bexarotene was superior to tamoxifen^13^. Tamoxifen is known to cause side effects in patients such as hot flashes and an increased risk of endometrial carcinoma^18^. SERMs are an effective treatment and prevention option for ER + breast cancer, but fewer strategies exist for hormone receptor negative breast cancers, despite high morbidity and mortality. With their enhanced efficacy coupled with a favorable toxicity profile, rexinoids may be useful not only for cancer treatment but also for prevention. In 2002, Wu et al. tested bexarotene for prevention in MMTV-erbB2 transgenic mice. Bexarotene significantly increased tumor-free survival, even at doses as low as 10 mg/kg^19^. The more potent rexinoid LG100268 and a SERM were highly effective at delaying tumor development in a similar murine model of HER2 + breast cancer, while the combination of a SERM and LG100268 completely prevented tumor development over a 50 week period of treatment^20^.

In addition to breast cancer, rexinoids are also effective in the A/J mouse model of lung cancer^21^. These mice develop *Kras* mutations in the lung after being challenged by carcinogens such as vinyl and ethyl carbamate, which form epoxides and DNA adducts, leading to the development of lung adenocarcinomas^22^. Activating mutations in *KRAS* are one of the most common oncogenic drivers in human lung adenocarcinomas^23^. Ethyl and vinyl carbamate are found in tobacco products^24^, making the A/J model an appropriate model of NSCLC in smokers. Several rexinoids inhibit growth of lung tumors in this model, including LG100268, LG101506, and IRX194204^25,26^.

Although rexinoids showed promising activity in several preclinical models of cancer^13,19,26^, clinical trials of bexarotene in both metastatic breast cancer and NSCLC did not lead to FDA approval. To improve clinical responses, new rexinoids with greater potency are needed. As rexinoids are also effective in cancer prevention studies, the toxicity profile of rexinoids must be monitored, as patients on long-term treatment with bexarotene experienced cumulative side effects, particularly elevated triglycerides and hypothyroidism^27^. To address these challenges, we synthesized novel analogues of bexarotene using molecular modeling software to guide chemical substitutions and rational drug design. These new compounds activated RXR with nanomolar potency and stimulated RXR-regulated transcription with minimal RAR activation^28,29^.

**Figure 1:**
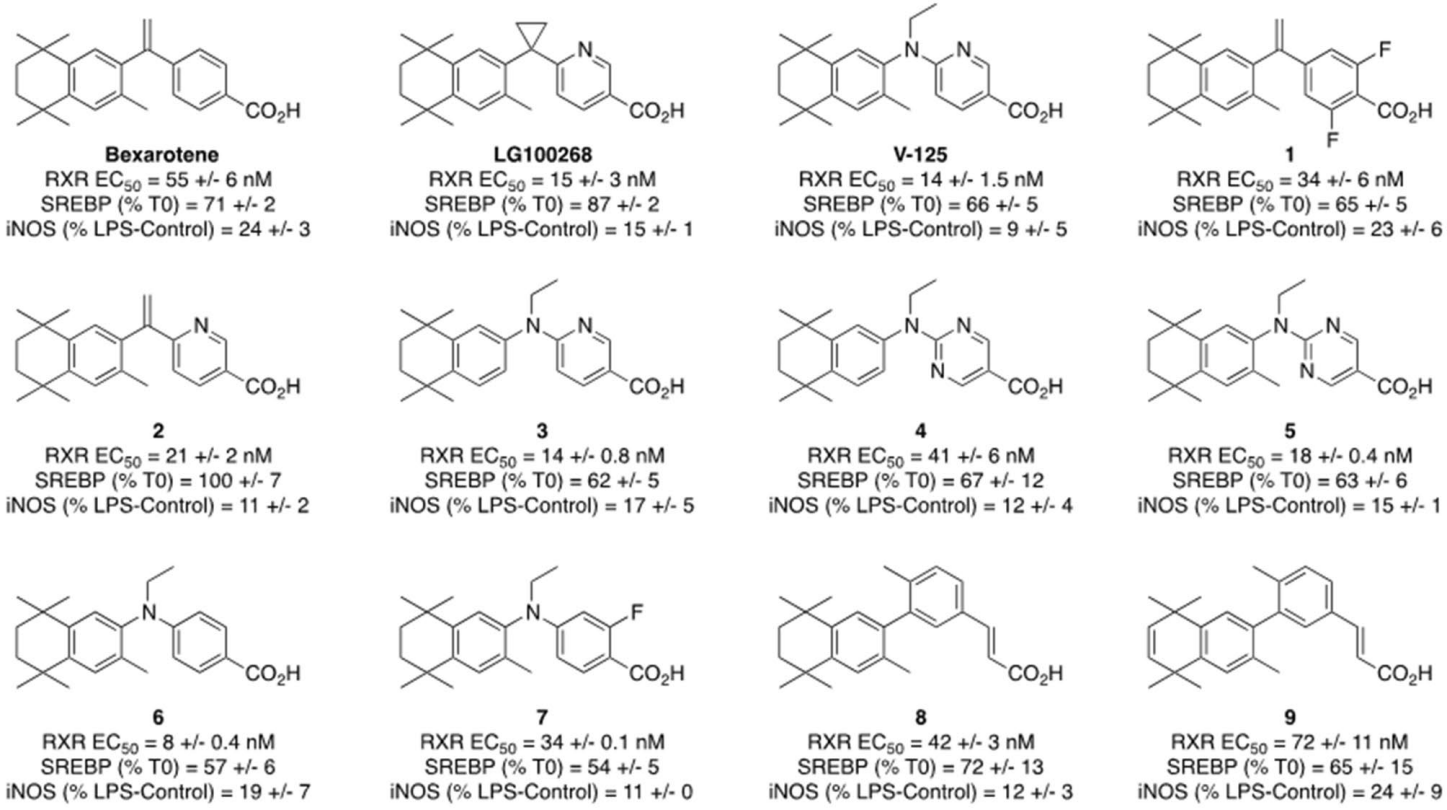
Structures and activities of bexarotene, LG100268, V-125 and nine rexinoids evaluated by PCA. Bexarotene is approved to treat cutaneous T-cell lymphoma, and V-125 was identified as a promising novel rexinoid based on its activity in published in vitro assays^30^. The iNOS suppression in RAW 264.7 cells was determined by cell treatment with 100 nM rexinoid followed by challenge with 1 ng/mL LPS over 24 h. Griess assay measured NO production normalized to LPS-stimulated control. SREBP and RXR activation were assessed as reported in prior work^30^. T0 is T0901317, a known LXR agonist and activator of SREBP.

To identify rexinoids with potential in vivo efficacy, we have developed a series of in vitro screening assays^30^. Of ten new rexinoids screened in these assays, V-125 (Fig. 1) was selected as the lead compound, as it was the most potent in the iNOS suppression assay, an assay which correlates with efficacy in the A/J model of lung cancer^30^. The EC_50_ value for RXRα activation for V-125 was 14 nM vs. 55 nM for bexarotene. Activation of sterol regulatory element-binding protein (SREBP), a biomarker of triglyceride elevation was lower with V-125 than either bexarotene or LG100268^30^. To further validate our in vitro screening paradigm, we tested the efficacy and safety of V-125 in preclinical models of breast and lung cancer.

## Materials and methods

### Drugs

Synthesis of V-125 was previously described^31^. Bexarotene was purchased from LC Laboratories. Rexinoids were dissolved in a vehicle (50 ml/kg diet) of 1 part ethanol:3 parts highly purified coconut oil (Neobee oil, Thermo Fisher) and then mixed into 1 kg of powdered diet (AIN-93M, BioServ for A/J mice or 5002 rodent chow, PMI Nutrition International for MMTV-neu mice) using a commercial KitchenAid mixer. The same vehicle was used in the control diets. In the MMTV-Neu model, doses were 30 mg per kg of diet (~ 7.5 mg per kg body weight) for prevention studies and 100 mg per kg of diet (~ 25 mg per kg body weight) for treatment studies, as utilized in previous publications^20,32^. In the A/J model, doses were 40 and 80 mg per kg of diet (~ 10 and 20 mg per kg body weight), doses used in previous publications^25^. Rexinoids remain stable in diet at 4 °C for up to 6 weeks, as confirmed by liquid chromatography-mass spectrometry.

### In vivo experiments

All animal studies were approved by the Institutional Animal Care and Use Committee at Michigan State University (IACUC protocol number 201800050). All protocols were carried out ethically in accordance with the Regulations for the Management of Laboratory Animals at Michigan State University and in compliance with the ARRIVE guidelines. Every effort was made to minimize suffering throughout these studies. Mice were euthanized by inhalation of carbon dioxide followed by cervical dislocation.

#### Lung carcinogenesis studies

Female A/J (Jackson Laboratory) mice at 7 and 8 weeks of age were injected intraperitoneally with vinyl carbamate (16 mg/kg, Toronto Research Chemicals) in a vehicle of isotonic saline. One week after the last injection of vinyl carbamate, rexinoids were fed in diet as described above. After 16 weeks of treatment, mice were euthanized and tissues collected. Lungs were inflated with PBS, and tumor number on the surface of the lungs was evaluated. The left lung was then fixed in 10% neutral buffered formalin; samples were blinded and randomized. The lung was sectioned and stained with H&E for evaluation of the histopathology on multiple sections, using previously established criteria^25^.

#### Breast cancer studies

For prevention studies, 10-week-old female MMTV-Neu mice^33^ (Jackson Laboratory) were fed control diet or 30 mg rexinoid per kg diet. Littermate-matched controls were used whenever possible. Mice were palpated twice weekly for the development of new tumors. For treatment studies, mice were fed standard diet and palpated twice weekly until tumors reached 5 mm in diameter, at which time mice were randomized and started on control diet or 100 mg V-125 per kg of diet. Mice were fed until tumors reached IACUC-defined endpoints (overall survival studies) or for 10 days (biomarker studies). At study conclusion, mice were euthanized, tissues were harvested, and tumors and livers weighed.

### Triglyceride quantification

Plasma was harvested from mice at necropsy. Triglyceride and cholesterol levels were measured using the Triglyceride Quantification Assay Kit (Abcam) or the Cholesterol Quantification Kit (Sigma-Aldrich) as per the manufacturers’ recommended protocols.

### Immunohistochemistry

Formalin-fixed tissues were embedded in paraffin and sectioned. Antigen retrieval was performed by boiling in citrate buffer, and endogenous peroxidase activity was quenched using hydrogen peroxide. Tissue sections were stained with antibodies against CD206 (1:200, Abcam), cleaved caspase 3 (1:100, 5A1E, Cell Signaling), PCNA (1:200, sc-56, Santa Cruz Biotechnologies), PD-L1 (1:50, MIH6, Abcam), and CD8 (1:40, 53–6.7, Biolegend) as described^32^. Sections were then labeled with biotinylated secondary antibodies (anti-rabbit, Cell Signaling; anti-rat, Vector Labs), as previously described^32^. Signal detection was performed using a DAB (3, 3'-diaminobenzidine) substrate (Cell Signaling). Sections were counterstained with hematoxylin (Vector Labs).

### Statistical analysis

Results were expressed as the means ± standard errors as indicated in figure legends. Kaplan–Meier survival curves from in vivo experiments were analyzed using the log rank test. Data from plasma triglyceride and cholesterol experiments were analyzed using one-way ANOVA, and significant differences between groups were determined by the Tukey HSD multiple comparison method (VassarStats.com). Data from lung experiments were analyzed by one-way ANOVA. When data fit a normal distribution, the Holm-Sidak test for multiple comparison was used. The Kruskal–Wallis one-way ANOVA on ranks followed by the Dunn test for multiple comparisons was used if the data did not pass the normality test. McNemar’s Z test was used for analysis of histopathological grades of lung tumors. *p* < 0.05 was considered statistically significant throughout all experiments.

### Principal component analysis (PCA) plot

Clustvis^34^ was used to analyze data and prepare PCA and heatmap plots as seen in Figs. S1 and S2 to analyze compounds for biological activity motifs. Ellipses in the PCA indicate 95% confidence intervals^35,36^.

### PCR array

Tumors from mice treated with V-125 (100 mg/kg diet) or control diet for 10 days were flash frozen, and total RNA was extracted using a RNeasy mini kit (Qiagen). DNA was eliminated with a RNase-free DNase kit (Qiagen). RNA purity and quality were assessed by Nanodrop and an Agilent 2100 Bioanalyzer. Quality of all samples exceeded the manufacturer’s recommendations. cDNA was synthesized using RT2 First Strand Kit (Qiagen), and samples were analyzed with the RT2 Profiler PCR Array (Mouse Breast Cancer, Qiagen) as per the manufacturer’s recommended protocols in the QuantStudio 6 Flex system with the following cycling conditions: 10 min at 95 °C, 15 s at 95 °C, 1 min 60 °C for 40 cycles.

### Quantitative PCR

qPCR was performed to validate mRNA expression of IL-6, Jun, and COX2 in tumors. mRNA was quantified using SYBR green, and values were normalized to the housekeeping gene GAPDH.

## Results

### V-125 clusters favorably in computational analyses of in vitro activity

We have found that applying principal component analysis (PCA) in our prior work^35,36^ with rexinoids has helped to visualize particularly active rexinoids with improved therapeutic potential, and to that end, we have conducted a PCA with V-125 and the nine other novel rexinoids (Fig. 1) to visualize where they group relative to each other as well as bexarotene and LG100268 (Figs. S1, S2). The PCA analysis resulted in three groups—A, B, and C—in which rexinoids clustered according to their activities in in vitro assays. In Group A, containing bexarotene, we observed the difluorobexarotene analog (**1**) as well as a rexinoid with a biphenyl structural motif (**9**). In Group B, containing LG100268, we observed a pyridine analog of bexarotene (**2**) as well as a pyrimidine containing rexinoid (**4**) and another biphenyl rexinoid (**8**). In group C, containing V-125, we observed the other rexinoids with structurally similar features—**3**, **5**, **6**, and **7**—though, V-125 was somewhat isolated whereas the other rexinoids in this group clustered more closely together. We envision PCA to be useful to quickly assess potential of novel rexinoids by seeing where they group according to their activities in these assays.

### V-125 prevents lung tumor development in A/J mice

**Figure 2:**
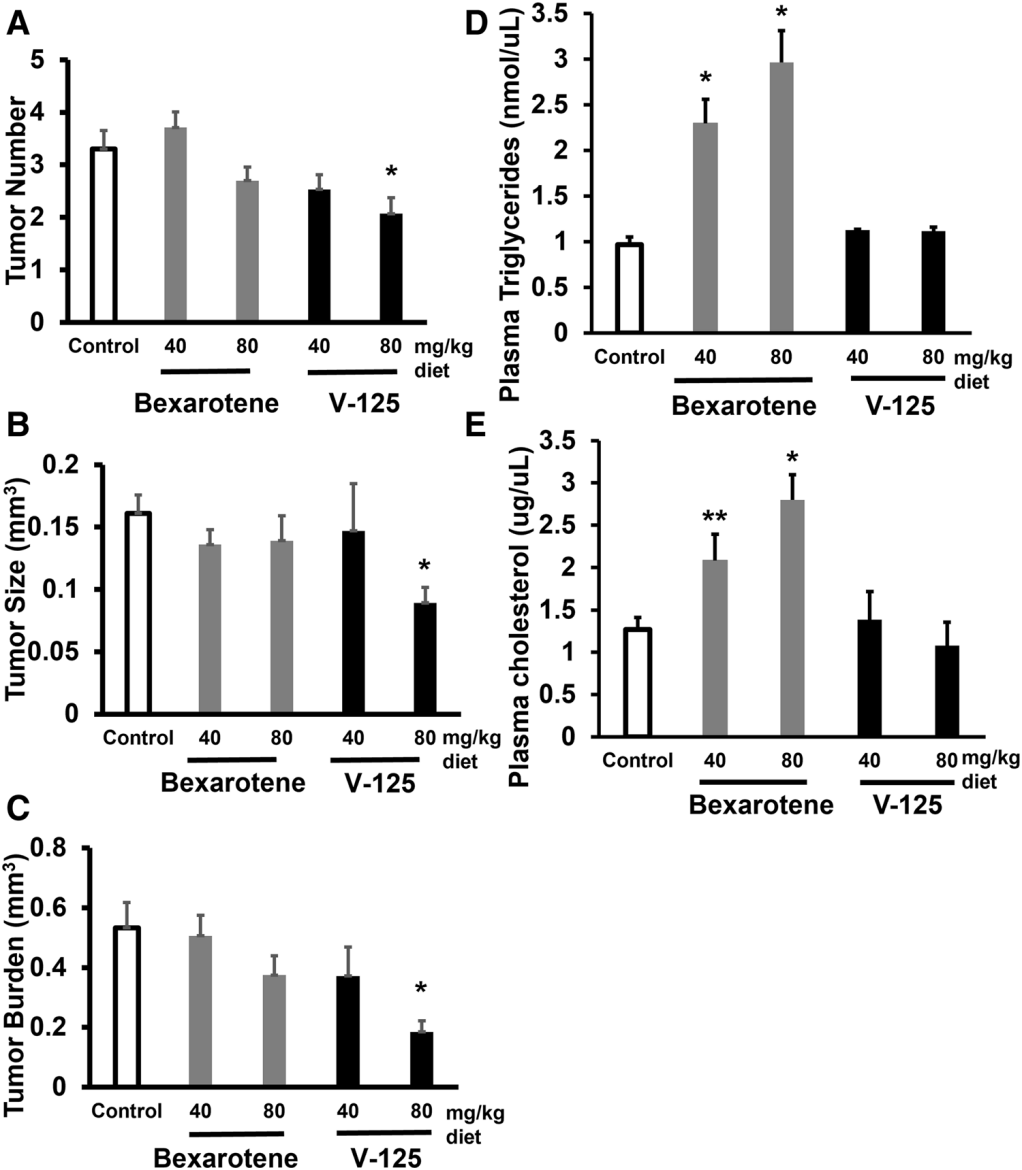
V-125 reduces lung tumor burden in A/J mice. Female A/J mice were injected with vinyl carbamate and starting 2 weeks later, fed control diet or V-125 in diet for 16 weeks. At the end of the study, mice were euthanized and lungs sectioned for analysis. Average tumor number (**A**), tumor size (**B**), and tumor burden (**C**) in control vs. drug treated groups. Results shown as means ± SE (*P < 0.05 vs. control). Control, bexarotene 40 and 80 mg/kg, and V-125 40 mg/kg: N = 15 mice per group. V-125 80 mg/kg: N = 14 mice per group. Plasma triglycerides (**D**) and cholesterol (**E**) were measured with commercial kits (*P < 0.01 vs. control; **P < 0.001 vs. control; n = 6 samples/group). Error bars represent standard deviations.

A/J mice were challenged with vinyl carbamate to induce *Kras* mutations and subsequent lung adenocarcinomas^37^ and then treated with V-125. Mice were fed control diet or rexinoids in diet starting two weeks post-carcinogen for 16 weeks. At the conclusion of the study, evaluation of lung tumor number, size, and histopathology on slides (2 per mouse) was done in a randomized and blinded manner by two independent investigators. V-125 at 80 mg/kg of diet significantly (p < 0.01, Fig. 2A–C) reduced tumor size by 45% (0.09 ± 0.01 mm^3^/tumor vs. 0.16 ± 0.01 mm^3^/tumor in the control group), tumor number by 37% (2.07 ± 0.30 vs. control 3.3 ± 0.35), and tumor burden by 65% (0.18 ± 0.04 mm^3^ vs. 0.53 ± 0.08 mm^3^ in the control group). While there was a trend toward lower tumor parameters in mice treated with bexarotene (Table 1), these changes were not statistically significant at either the 40 or 80 mg/kg doses.

**Table 1:** V-125 reduces lung carcinogenesis in A/J mice.

mg/kg diet	Control	Bexarotene		V-125	
		40	80	40	80
# of slides/group	30	28	30	30	28
# of tumors/group	99	104	81	76	58
Total # tumors/slide (% control)	3.3 ± 0.35 (100%)	3.71 ± 0.30 (112.6%)	2.7 ± 0.26 (81.8%)	2.53 ± 0.28 (76.8%)	2.07 ± 0.30* (62.8%)
Total tumor volume, mm^3^	15.98	14.17	11.247	11.16	5.17
Ave tumor size (mm^3^)/tumor (% control)	0.16 ± 0.01 (100%)	0.14 ± 0.01 (84.4%)	0.14 ± 0.02 (84.4%)	0.15 ± 0.04 (91.0%)	0.09 ± 0.01* (55.2%)
Ave tumor burden (mm^3^) (% control)	0.53 ± 0.08 (100%)	0.51 ± 0.07 (95.0%)	0.37 ± 0.06 (70.4%)	0.37 ± 0.10 (69.8%)	0.18 ± 0.04* (34.7%)
Total # L/M grade (% total)	43 (43%)	50 (48%)	43 (53%)	38 (50%)	38 (66%)*
Total # HH grade (% total)	56 (57%)	54 (52%)	38 (47%)	38 (50%)	20 (34%)*

Female A/J mice were injected with vinyl carbamate and fed control diet or V-125 in diet for 16 weeks, as described in Fig. 2.

*L* low, *M* medium, *H* high.

*P < 0.05 vs. control.

Although both V-125 and bexarotene were well-tolerated in this model based on animal weights (Fig. S3A), bexarotene elevates triglycerides in animal models^21^ and in human patients^38^. As shown in Fig. 2D, bexarotene significantly (p < 0.01) increased plasma triglyceride levels at both the 40 and 80 mg/kg doses (2.30 ± 0.26 nmol/μL and 2.97 ± 0.35 nmol/μL, respectively, vs. control 0.97 ± 0.09 nmol/μL) in the A/J mice. In contrast, V-125 had no effect on plasma triglycerides. Bexarotene also significantly (p < 0.001) elevated plasma cholesterol (Fig. 2E) in a dose-dependent manner (2.08 ± 0.31 µg/μL in mice fed bexarotene at 40 mg/kg of diet and 2.80 ± 0.30 µg/μL in mice fed bexarotene at 80 mg/kg of diet, vs control 1.27 ± 0.14 µg/μL), while V-125 did not significantly increase plasma cholesterol at either dose.

Lung tumors were classified as low, medium, or high grade based on previously established criteria^25^ (Table 1). Treatment with 80 mg/kg V-125 significantly (p < 0.05) increased the proportion of tumors graded low/medium (66% vs. control 43%) and reduced (p < 0.05) the proportion of tumors classified as high grade (34% vs. control 57%). In comparison, bexarotene did not significantly change the proportions of tumors of any grade at either dose.

### V-125 delays the development of HER2-positive mammary tumors in MMTV-Neu mice

**Figure 3:**
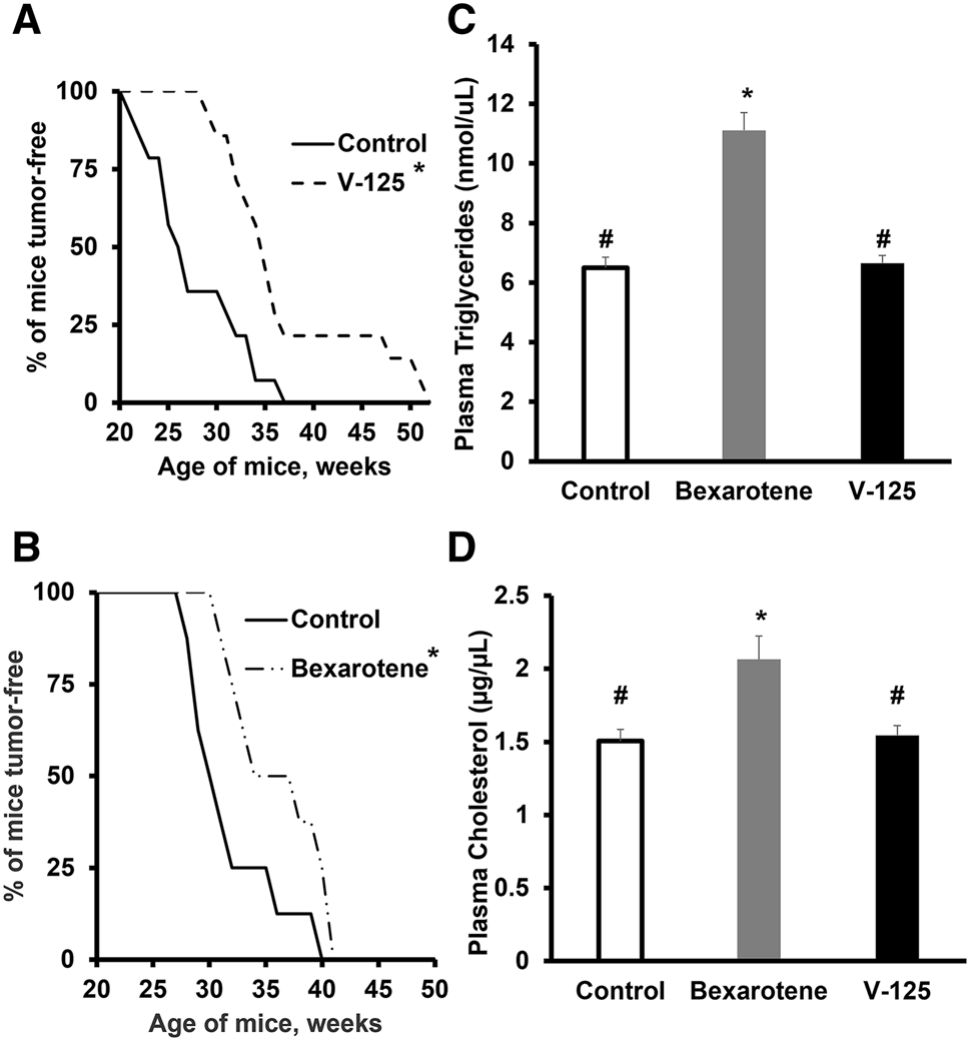
V-125 delays development of mammary tumor in MMTV-Neu mice. 10 week old female MMTV-Neu mice were fed control diet or 30 mg/kg diet of the rexinoids V-125 (**A**) or bexarotene (**B**). Mice were palpated twice weekly for the development of new tumors. (**A**) *P < 0.001 vs. control via log rank test; N = 14 mice per group. (**B**) *P < 0.05 vs. control via log rank test; N = 8–9 mice per group. Plasma triglycerides (**C**) and cholesterol (**D**) levels in mice treated with control or rexinoids (30 mg/kg diet) were quantified using commercial kits (*P < 0.01 vs. control; ^#^P < 0.01 vs. bexarotene; n = 8 mice/group).

MMTV-Neu mice express wild-type, unactivated Neu in mammary tissue under the control of the mouse mammary tumor virus (MMTV) promoter^33^ and develop focal mammary adenocarcinomas by 25–35 weeks of age^39^. To investigate the chemopreventive effects of V-125 in this preclinical model of HER2 + breast cancer^40,41^, MMTV-neu mice were fed control diet or V-125 in diet (30 mg/kg) starting at 10 weeks of age. V-125 significantly (p < 0.001) delayed initial tumor development compared to the control group (Fig. 3A), resulting in an approximately 10 week increase in mean time to tumor development (36.1 ± 7.8 weeks vs. control 26.5 ± 4.9 weeks). Treatment with V-125 was well tolerated in this study (Fig. S3B). Bexarotene also significantly (p < 0.05) increased the time to initial tumor development in MMTV-neu mice (Fig. 3B) but was not as effective as V-125, delaying tumor development by only 4 weeks (35.9 ± 4.2 weeks vs. control 31.7 ± 4.1 weeks).

Plasma triglycerides and cholesterol levels were measured at the end of this study (Fig. 3C,D). Bexarotene significantly (p < 0.01) increased plasma triglyceride levels (11.1 ± 1.7 nmol/μL vs. control 6.5 ± 0.98 nmol/μL). In comparison, V-125 did not elevate plasma triglycerides (6.6 ± 0.73 nmol/μL). Bexarotene also significantly (p < 0.01) raised plasma cholesterol levels (Fig. 3D) (2.1 ± 0.16 µg/μL vs control 1.5 ± 0.08 µg/μL), while V-125 had no effect.

### Treatment with V-125 extends overall survival in MMTV-Neu mice

**Figure 4:**
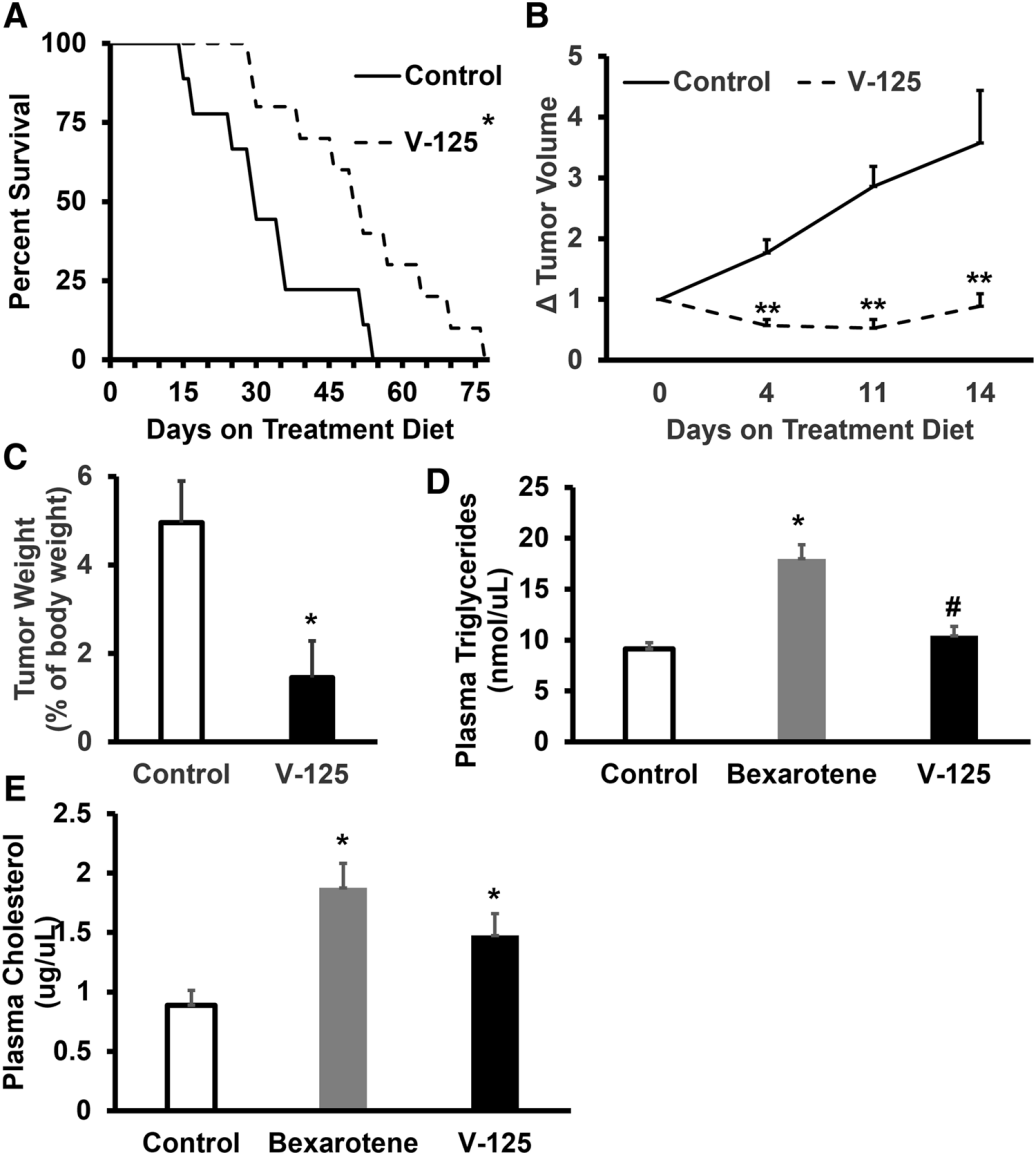
V-125 extends overall survival in MMTV-Neu mice. Female MMTV-Neu mice with established mammary tumors 5 mm in diameter were fed control diet or V-125 (100 mg/kg in diet) until tumors reached endpoint as defined by IACUC guidelines (**A,B**) or for 10 days (**C**–**E**). (**A**) Overall survival is plotted for control and V-125-treated groups (*P < 0.0172 vs. control via log rank test; N = 9–10 mice per group). (**B**) Tumor volume over the first 14 days of treatment with V-125 was calculated and normalized to initial tumor volume (**P < 0.01 vs. control). **C.** Tumor weight at the end of 10 days of treatment is presented as a percent of body weight (*P < 0.05 vs. control; N = 6–8 mice per group). Plasma triglycerides (**D**) and cholesterol (**E**) levels in mice treated with control or rexinoids (100 mg/kg diet) for 10 days were quantified using commercial kits (*P < 0.01 vs. control; ^#^P < 0.01 vs. bexarotene; n = 6–11 mice/group). All error bars represent the standard errors.

To evaluate the anti-tumor efficacy of V-125, MMTV-Neu mice with established tumors measuring 5 mm in diameter were treated with control diet or V-125 at a dose of 100 mg/kg of diet. Tumors were measured twice weekly until they reached 10 mm in diameter, at which point they were euthanized per IACUC guidelines. Time from initiation of drug diet to euthanasia was compared between groups. Treatment with V-125 significantly (p < 0.0172) increased overall survival (51.6 ± 15.8 days vs. control 32.8 ± 13.6 days) (Fig. 4A). Average tumor volume over the first 14 days of treatment was calculated and normalized to the initial tumor volume when treatment diet was started. The average change in tumor volume of the V-125-treated group was significantly (p < 0.01) lower than the average tumor volume in the control group at day 4 (0.57 ± 0.10 vs. control 1.8 ± 0.22), day 11 (0.53 ± 0.14 vs. control 2.9 ± 0.33), and day 14 (0.89 ± 0.21 vs. control 3.6 ± 0.86) of treatment (Fig. 4B).

To identify biomarkers of V-125 efficacy, MMTV-Neu mice with established tumors were treated with 100 mg V-125/kg diet for 10 days. A treatment duration of 10 days was selected based on the drastic decrease in tumor volume observed on days 10–11 of treatment (Fig. 4B). As shown in Fig. 4C, V-125 significantly (p < 0.05) decreased tumor weight, presented as a percentage of total body weight (1.46 ± 0.82% vs. control 4.95 ± 0.95%). Ten days of treatment with 100 mg bexarotene/kg diet (Fig. S4) also significantly (p < 0.05) decreased tumor weight (2.52 ± 0.41% of total body weight vs. control 3.78 ± 0.49%), but only by 1.26%, nearly three times less than the effect observed with V-125.

When triglyceride levels in MMTV-Neu mice were evaluated, treatment with 100 mg bexarotene/kg diet for 10 days significantly (p < 0.01) elevated plasma triglyceride levels (18.0 ± 1.4 nmol/μL vs. control 9.1 ± 0.61 nmol/μL). In contrast, V-125 did not change plasma triglycerides (10.4 ± 2.3 nmol/μL vs. control 9.12 ± 0.61 nmol/μL), and the triglyceride levels observed in mice treated with V-125 were significantly (p < 0.01) lower than those in bexarotene-treated mice (Fig. 4D). Both bexarotene and V-125 significantly increased plasma cholesterol at this dose (1.87 ± 0.21 µg/μL and 1.47 ± 0.18 µg/μL respectively, vs. control 0.89 ± 0.12 µg/μL; Fig. 4E).

### V-125 alters immune-related biomarkers in MMTV-Neu tumors

**Figure 5:**
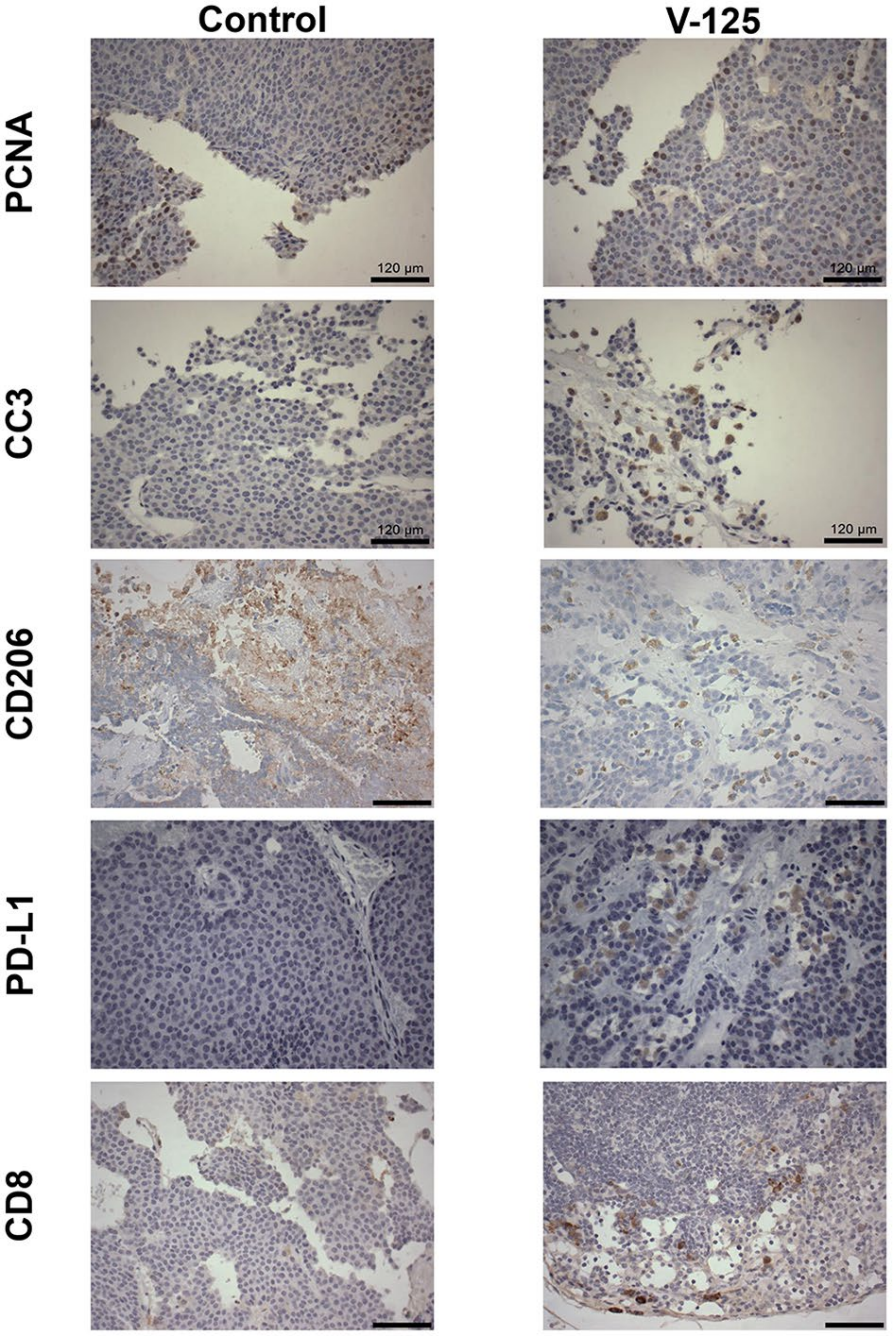
V-125 alters cell surface and intracellular markers in the MMTV-neu model of breast cancer. Mice with established mammary tumors 5 mm in diameter were treated with control or V-125 (100 mg/kg in diet) for 10 days. Immunohistochemical staining was performed for PCNA (proliferation), cleaved caspase-3 (CC3, apoptosis), CD206 (tumor-associated macrophages), PD-L1 (immune checkpoint), and CD8 (cytotoxic T cells) in the tumors. Scale bar represents 120 μm.

MMTV-Neu tumors were harvested from mammary glands after 10 days of treatment with V-125 and sectioned for immunohistochemistry (Fig. 5). In these tumors, V-125 did not change expression of proliferating cell nuclear antigen (PCNA), a marker of cell proliferation. However, there was a marked increase in cleaved caspase 3, a marker of apoptosis, in tumors treated with V-125. As previous studies have demonstrated the immunomodulatory effects of rexinoids^32^, we also examined the expression of CD206 and programmed death-ligand 1 (PD-L1) in tumors. Tumors of mice treated with V-125 had a striking decrease in CD206, a cell surface marker expressed by immunosuppressive macrophages. V-125 also increased expression of PD-L1, an immune checkpoint molecule, and CD8, a marker of cytotoxic T cells.

### V-125 differentially regulates gene expression in tumors of MMTV-Neu mice

**Figure 6:**
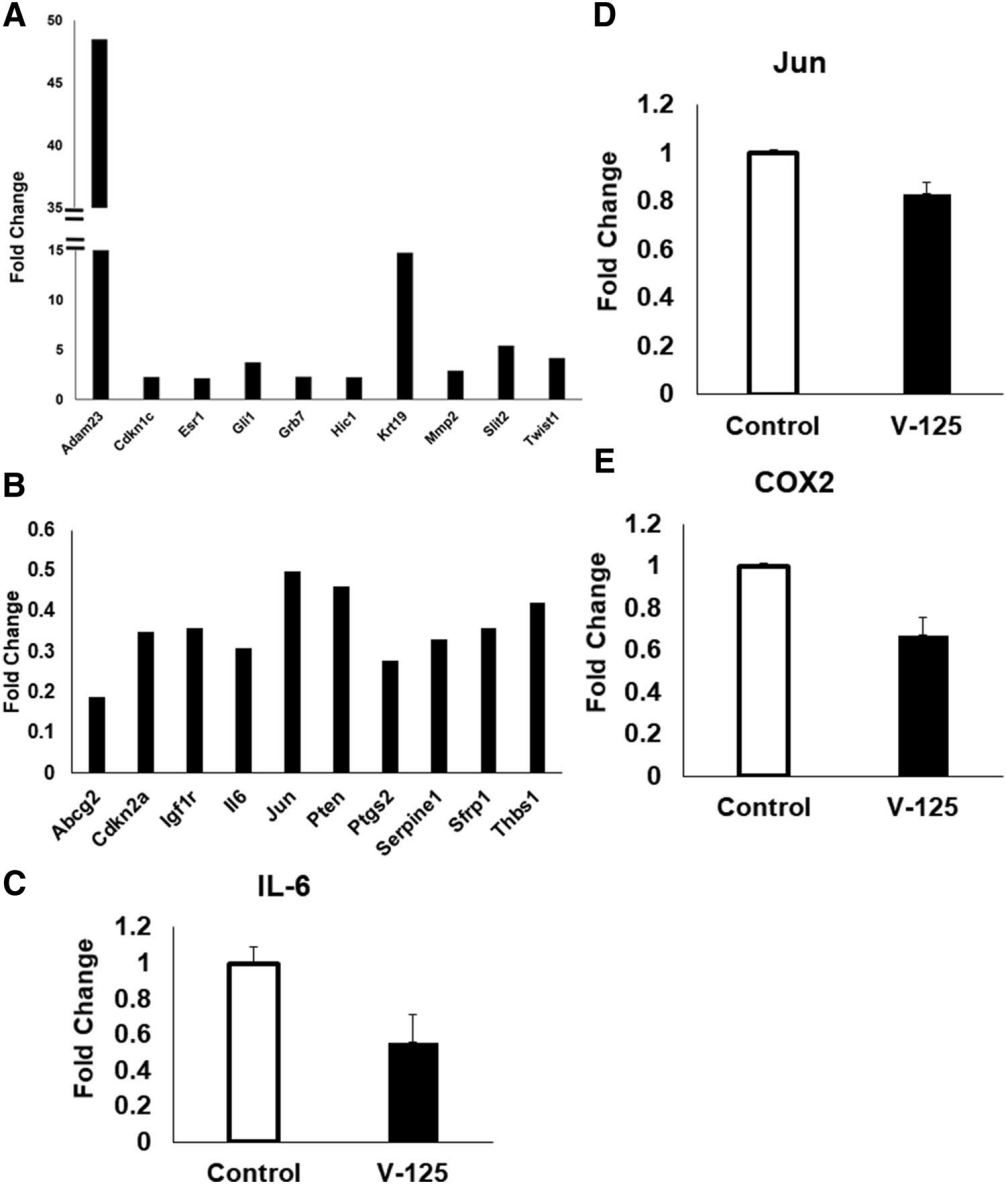
V-125 differentially regulates cancer-associated genes in tumors. Mice with established mammary tumors 5 mm in diameter were treated with control or V-125 (100 mg/kg in diet) for 10 days. Total RNA was extracted for PCR array. Analysis was performed using GeneGlobe software (Qiagen), revealing the upregulation of 10 genes with a log fold change > 2 (**A**), and the downregulation of 10 genes with a log fold change > 2 (**B**). Validation was performed on IL-6 (**C**), Jun (**D**), and COX2 (**E**) by qPCR. N = 2 mice/group. Data shown is representative of two independent experimental runs.

MMTV-Neu tumors were harvested after 10 days of treatment with V-125 (100 mg/kg) or control diet. A PCR array (Fig. 6) revealed upregulation of the following genes in the V-125 group with a log fold change > 2: *Adam23, Cdkn1c, Esr1, Gli1, Grb7, Hic1, Krt19, Mmp2, Slit2,* and *Twist1* (Fig. 6A). The following genes were downregulated by treatment with V-125, again with a log fold change > 2: *Abcg2, Cdkn2a, Igf1r, IL-6, Jun, Pten, Ptgs2, Serpine1, Sfrp1,* and *Thbs1* (Fig. 6B). To validate these results, qPCR for *IL-6* (Fig. 6C), *Jun* (Fig. 6D), and *COX2* (Fig. 6E) mRNA using tumor RNA from 2 mice per group confirmed the decreased expression of these genes in tumors from mice treated with V-125 in comparison to the controls.

## Discussion

Our results demonstrate significant efficacy of the novel rexinoid V-125 for prevention and treatment in the MMTV-Neu model of HER2 + breast cancer and prevention in the A/J model of Kras-driven NSCLC. Notably, V-125 was more efficacious than the clinically approved rexinoid bexarotene in both models. Unlike bexarotene, V-125 did not elevate plasma triglycerides, although it did elevate plasma cholesterol levels at its highest treatment dose (100 mg/kg diet) in the MMTV-Neu model. The combination of improved efficacy and reduced toxicity in two different clinically relevant animal models suggests that V-125 is a strong candidate for further development toward a new therapy for the treatment of patients with breast or lung cancer.

These studies also provide proof of principle that more effective and better tolerated rexinoids can be developed through structural modifications to existing rexinoids and further validate the in vitro screening paradigm we previously optimized. These in vitro assays demonstrated that V-125 does not elevate SREBP, a transcription factor which regulates genes responsible for lipid homeostasis (Fig. 1).

Elevation of SREBP in vitro predicts for increased plasma cholesterol and triglycerides in vivo^21,30^. Furthermore, V-125 has anti-inflammatory activity in the iNOS suppression assay at the nM level (Fig. 1), which correlates with efficacy for prevention in the A/J lung cancer model, and nM activity in a RXRα reporter assay^30^. As predicted by our in vitro screening assays, V-125 treatment resulted in superior anti-tumor efficacy without elevating triglycerides as observed with bexarotene. This screening paradigm was also used to select the compound MSU42011 which, as predicted based on the in vitro screening assays, reduced lung tumor burden in A/J mice^21^.

Rexinoids have been used clinically for both treatment (NCT00003752 and NCT04664829) and prevention (NCT00055991 and NCT03323658) of breast cancer. As expected, lower doses are needed for prevention studies (30 mg/kg diet in the MMTV-neu model and 40–80 mg/kg diet in the more aggressive A/J model of lung cancer) than treatment studies (100 mg/kg in the MMTV-Neu model), as early intervention is more amenable to pharmacological intervention than treating late-stage, advanced disease^42^. A compound must be very well-tolerated to warrant long-term prevention studies in patients, and lower doses will help reduce toxicity. In comparison, higher doses are used for treatment, as larger tumors have acquired multiple mutations and are more resistant to drugs. Importantly, with the combination of high anti-tumor efficacy and a favorable safety profile, rexinoids may be utilized in either clinical setting^27^. While recent strategies utilizing novel aerosolized delivery systems for administration of bexarotene have been effective in rodent models of lung cancer without inducing hypertriglyceridemia or hypercholesterolemia^43,44^, it is prudent to continue to synthesize new RXR agonists with improved efficacy and toxicity profiles in addition to optimizing drug delivery strategies. A combination of these two strategies may yield the best results.

The mechanism of action of V-125 is complex. V-125 had a limited effect on proliferation of tumor cells, visualized by PCNA expression (Fig. 5). However, V-125 induced apoptosis in MMTV-Neu tumors as evidenced by the increased expression of cleaved caspase 3. Tumor size is dependent on the balance between cancer cell growth and death^45^, therefore despite a limited effect on cell proliferation, V-125 efficaciously reduced tumor size by inducing apoptosis. Rexinoids have limited ability to induce apoptosis of cancer cells *in* vitro^32^, leading to the hypothesis that immune cells in the tumor microenvironment play a significant role in mediating the anti-tumor efficacy of rexinoids observed in vivo. Previous studies from Leal et al. investigated the immunomodulatory effects of the rexinoids LG100268 and bexarotene and noted significant changes in T cell populations and activation markers only with LG100268^32^. Zhang et al. detected rexinoid-induced changes in the phenotype and function of macrophages in the tumor microenvironment^30^, which may contribute to anti-tumor efficacy^46^. These data, combined with the iNOS screening data in RAW 264.7 macrophage-like cells^30^, suggest that the effects of V-125 on macrophages may be necessary for anti-tumor activity. Indeed, the reduction in CD206 staining evident in MMTV-Neu tumors treated with V-125 (Fig. 5) confirms that this compound reduces populations of immunosuppressive macrophages associated with tumor progression. There are several possible explanations for this reduction: V-125 treatment may result in macrophage skewing, altering the polarization of these cells away from a tumor-promoting phenotype and towards a tumor-suppressive phenotype^47^. Alternatively, this decrease in CD206 staining in mice treated with V-125 could be the result of altered immune cell infiltration into the tumor, a change in the localization of immunosuppressive macrophages, or systemic effects on monocyte differentiation or circulation. Notably, we have previously demonstrated that novel pyrimidinyl derivative of LG100268 decreased F4/80 + lung macrophage populations in A/J mice^30^. Further studies are needed to determine how V-125 exerts these immunomodulatory effects in these models and how critical these effects are for anti-tumor efficacy.

The increased PD-L1 expression in tumors treated with V-125 provides an intriguing potential avenue for the combination of rexinoids with immunotherapy. The expression of PD-L1 is a positive prognostic marker in breast cancer, indicating a hot immune microenvironment^48^. In basal tumors, upregulation of PD-L1 correlates with strong local cytotoxic T cell responses and positive responses to neoadjuvant chemotherapy^48^. High PD-L1 expression and increased numbers of tumor-infiltrating lymphocytes may be predictive of patient populations that would benefit from immunotherapy^49^. As V-125 increased the expression of PD-L1 in tumors, this may bolster response to immunotherapies such as atezolizumab, a monoclonal antibody which selectively targets PD-L1^50^. Combination treatment with rexinoids and immunotherapy is a promising area for further investigation.

A breast cancer PCR array revealed differentially regulated genes in mammary tumors treated with V-125. *Adam23*, which is increased in V-125 tumors compared to controls, is a cellular adhesion molecule that enhances progression of breast cancer when downregulated^55^. *Slit2* expression in breast cancer inhibits migration^56^ and is inactivated in both breast and lung cancer^57^. Importantly, *IL-6* expression was lower in V-125-treated tumors, suggesting that V-125 plays a role in modulating the tumor immune compartment. This finding was confirmed by qPCR and recapitulates our previous studies with other rexinoids^58^. Future studies will explore the role of these gene products in mediating the anti-tumor activity of V-125.

In conclusion, the novel rexinoid V-125 is an effective treatment in murine models of both breast cancer and lung cancer. The favorable toxicity profile of this compound allows for use in either cancer treatment or prevention.

## Supplementary Information

Supplementary Information.

## Data Availability

The datasets generated during and analyzed during the current study are available from the corresponding author on reasonable request.

## References

[1] Esfahani K (2020). A review of cancer immunotherapy: from the past, to the present, to the future. Curr Oncol..

[2] Pohlmann PR, Mayer IA, Mernaugh R (2009). Resistance to trastuzumab in breast cancer. Clin. Cancer Res..

[3] Kim ES (2016). Chemotherapy resistance in lung cancer. Adv. Exp. Med. Biol..

[4] Lin JJ, Shaw AT (2016). Resisting resistance: Targeted therapies in lung cancer. Trends Cancer.

[5] Evans RM, Mangelsdorf DJ (2014). Nuclear receptors, RXR, and the big bang. Cell.

[6] Leal, A. S., Reich, L. A., Moerland, J. A., Zhang, D. & Liby, K. T. in *Adv. Pharmacol.* Vol. 91 (eds Bryan L. Copple & Cheryl E. Rockwell) 141–183 (Academic Press, 2021). 10.1016/bs.apha.2021.01.004 34099107

[7] Hurst RE (2000). Bexarotene ligand pharmaceuticals. Curr. Opin. Investig. Drugs.

[8] Esteva FJ (2003). Multicenter phase II study of oral bexarotene for patients with metastatic breast cancer. J. Clin. Oncol..

[9] Khuri FR (2001). Multi-institutional phase I/II trial of oral bexarotene in combination with cisplatin and vinorelbine in previously untreated patients with advanced non-small-cell lung cancer. J. Clin. Oncol..

[10] Michellys PY (2003). Design and synthesis of novel RXR-selective modulators with improved pharmacological profile. Bioorg. Med. Chem. Lett..

[11] de Almeida NR, Conda-Sheridan M (2019). A review of the molecular design and biological activities of RXR agonists. Med. Res. Rev..

[12] Chan MM, Lu X, Merchant FM, Iglehart JD, Miron PL (2005). Gene expression profiling of NMU-induced rat mammary tumors: Cross species comparison with human breast cancer. Carcinogenesis.

[13] Gottardis MM (1996). Chemoprevention of mammary carcinoma by LGD1069 (Targretin): An RXR-selective Ligand. Cancer Res..

[14] Anzano MA (1994). Prevention of breast cancer in the rat with 9-cis-retinoic acid as a single agent and in combination with tamoxifen. Cancer Res..

[15] Teplitzky SR (2001). Chemoprevention of NMU-induced rat mammary carcinoma with the combination of melatonin and 9-cis-retinoic acid. Cancer Lett..

[16] Apfel C (1992). A retinoic acid receptor alpha antagonist selectively counteracts retinoic acid effects. Proc. Natl. Acad. Sci..

[17] Miller VA (1997). Initial clinical trial of a selective retinoid X receptor ligand, LGD1069. J. Clin. Oncol..

[18] Jordan VC (1995). Alternate antiestrogens and approaches to the prevention of breast cancer. J. Cell Biochem. Suppl..

[19] Wu K (2002). The retinoid X receptor-selective retinoid, LGD1069, prevents the development of estrogen receptor-negative mammary tumors in transgenic mice. Cancer Res..

[20] Liby K (2006). The combination of the rexinoid, LG100268, and a selective estrogen receptor modulator, either arzoxifene or acolbifene, synergizes in the prevention and treatment of mammary tumors in an estrogen receptor-negative model of breast cancer. Clin. Cancer Res..

[21] Moerland JA (2020). The novel rexinoid MSU-42011 is effective for the treatment of preclinical Kras-driven lung cancer. Sci. Rep..

[22] Forkert P-G (2010). Mechanisms of lung tumorigenesis by ethyl carbamate and vinyl carbamate. Drug Metab. Rev..

[23] El Osta B (2019). Characteristics and outcomes of patients with metastatic KRAS-mutant lung adenocarcinomas: The lung cancer mutation consortium experience. J. Thorac. Oncol..

[24] Schmeltz I, Chiong KG, Hoffmann D (1978). Formation and determination of ethyl carbamate in tobacco and tobacco smoke. J. Anal. Toxicol..

[25] Liby K (2009). Triterpenoids CDDO-methyl ester or CDDO-ethyl amide and rexinoids LG100268 or NRX194204 for prevention and treatment of lung cancer in mice. Cancer Prev. Res..

[26] Cao M (2016). The rexinoids LG100268 and LG101506 inhibit inflammation and suppress lung carcinogenesis in A/J mice. Cancer Prev. Res..

[27] Uray IP, Dmitrovsky E, Brown PH (2016). Retinoids and rexinoids in cancer prevention: from laboratory to clinic. Semin. Oncol..

[28] Jurutka PW (2013). Modeling, synthesis, and biological evaluation of potential retinoid X receptor (RXR) selective agonists: novel analogues of 4-[1-(3,5,5,8,8-pentamethyl-5,6,7,8-tetrahydro-2-naphthyl)ethynyl]benzoic acid (bexarotene) and (E)-3-(3-(1,2,3,4-tetrahydro-1,1,4,4,6-pentamethylnaphthalen-7-yl)-4-hydroxyphenyl)acrylic acid (CD3254). J. Med. Chem..

[29] Wagner CE (2009). Modeling, synthesis and biological evaluation of potential retinoid X receptor (RXR) selective agonists: Novel analogues of 4-[1-(3,5,5,8,8-pentamethyl-5,6,7,8-tetrahydro-2-naphthyl)ethynyl]benzoic acid (Bexarotene). J. Med. Chem..

[30] Zhang D (2019). Testing novel pyrimidinyl rexinoids: a new paradigm for evaluating rexinoids for cancer prevention. Cancer Prev. Res..

[31] Heck MC (2016). Modeling, Synthesis, and Biological Evaluation of Potential Retinoid X Receptor (RXR)-Selective Agonists: Analogues of 4-[1-(3,5,5,8,8-Pentamethyl-5,6,7,8-tetrahydro-2-naphthyl)ethynyl]benzoic Acid (Bexarotene) and 6-(Ethyl(5,5,8,8-tetrahydronaphthalen-2-yl)amino)nicotinic Acid (NEt-TMN). J. Med. Chem..

[32] Leal AS (2019). Retinoid X receptor agonist LG100268 modulates the immune microenvironment in preclinical breast cancer models. NPJ Breast Cancer.

[33] Guy CT (1992). Expression of the neu protooncogene in the mammary epithelium of transgenic mice induces metastatic disease. Proc. Natl. Acad. Sci..

[34] Metsalu T, Vilo J (2015). ClustVis: a web tool for visualizing clustering of multivariate data using principal component analysis and heatmap. Nucleic Acids Res..

[35] Hanish BJ (2018). A novel gene expression analytics-based approach to structure aided design of rexinoids for development as next-generation cancer therapeutics. Steroids.

[36] Mallick S (2021). Evaluating novel RXR agonists that induce ApoE and tyrosine hydroxylase in cultured human glioblastoma cells. ACS Chem. Neurosci..

[37] Hernandez LG, Forkert PG (2009). Inhibition of vinyl carbamate-induced lung tumors and Kras2 mutations by the garlic derivative diallyl sulfone. Mutat. Res..

[38] Duvic M (2001). Bexarotene is effective and safe for treatment of refractory advanced-stage cutaneous t-cell lymphoma: Multinational phase II-III trial results. J. Clin. Oncol..

[39] Fry EA, Taneja P, Inoue K (2016). Clinical applications of mouse models for breast cancer engaging HER2/neu. Integr. Cancer Sci. Ther..

[40] Wong AW, Dunlap SM, Holcomb VB, Nunez NP (2012). Alcohol promotes mammary tumor development via the estrogen pathway in estrogen receptor alpha-negative HER2/neu mice. Alcohol. Clin. Exp. Res..

[41] Khazal KF, Hill DL, Grubbs CJ (2014). Effect of Withania somnifera root extract on spontaneous estrogen receptor-negative mammary cancer in MMTV/Neu mice. Anticancer Res..

[42] Sporn MB, Liby KT (2013). A mini-review of chemoprevention of cancer—past, present, and future. Prog. Chem..

[43] Zhang Q (2011). Aerosolized bexarotene inhibits lung tumorigenesis without increasing plasma triglyceride and cholesterol levels in mice. Cancer Prev. Res..

[44] Zhang Q (2019). Optimized bexarotene aerosol formulation inhibits major subtypes of lung cancer in mice. Nano Lett..

[45] Mommers ECM, van Diest PJ, Leonhart AM, Meijer CJLM, Baak JPA (1999). Balance of cell proliferation and apoptosis in breast carcinogenesis. Breast Cancer Res. Treat..

[46] Mantovani A, Marchesi F, Malesci A, Laghi L, Allavena P (2017). Tumour-associated macrophages as treatment targets in oncology. Nat. Rev. Clin. Oncol..

[47] Najafi M (2019). Macrophage polarity in cancer: A review. J. Cell. Biochem..

[48] Baptista MZ, Sarian LO, Derchain SFM, Pinto GA, Vassallo J (2016). Prognostic significance of PD-L1 and PD-L2 in breast cancer. Hum. Pathol..

[49] Wimberly H (2015). PD-L1 expression correlates with tumor-infiltrating lymphocytes and response to neoadjuvant chemotherapy in breast cancer. Cancer Immunol. Res..

[50] Schmid P (2018). Atezolizumab and nab-paclitaxel in advanced triple-negative breast cancer. N. Engl. J. Med..

[51] Simanshu DK, Nissley DV, McCormick F (2017). RAS proteins and their regulators in human disease. Cell.

[52] Wood K, Hensing T, Malik R, Salgia R (2016). Prognostic and Predictive value in KRAS in non-small-cell lung cancer: A review. JAMA Oncol..

[53] von Lintig FC (2000). Ras activation in human breast cancer. Breast Cancer Res. Treat..

[54] Banys-Paluchowski M (2020). Clinical relevance of H-RAS, K-RAS, and N-RAS mRNA expression in primary breast cancer patients. Breast Cancer Res. Treat..

[55] Costa FF (2004). Epigenetic silencing of the adhesion molecule ADAM23 is highly frequent in breast tumors. Oncogene.

[56] Schmid BC (2007). The neuronal guidance cue Slit2 induces targeted migration and may play a role in brain metastasis of breast cancer cells. Breast Cancer Res. Treat..

[57] Dallol A (2002). SLIT2, a human homologue of the Drosophila Slit2 gene, has tumor suppressor activity and is frequently inactivated in lung and breast cancers. Can. Res..

[58] Liby K (2008). Prevention and treatment of experimental estrogen receptor-negative mammary carcinogenesis by the synthetic triterpenoid CDDO-methyl Ester and the rexinoid LG100268. Clin. Cancer Res..

