# Supplementary material for: The rexinoid V-125 reduces tumor growth in preclinical models of breast and lung cancer
Source: Sci Rep. 2022 Jan 7;12:293. doi: 10.1038/s41598-021-04415-0 (PMC8742020; doi:10.1038/s41598-021-04415-0)
Supplement: Supplementary file 1 — Supplementary Information. [file 41598_2021_4415_MOESM1_ESM.pptx]

## Slide 1
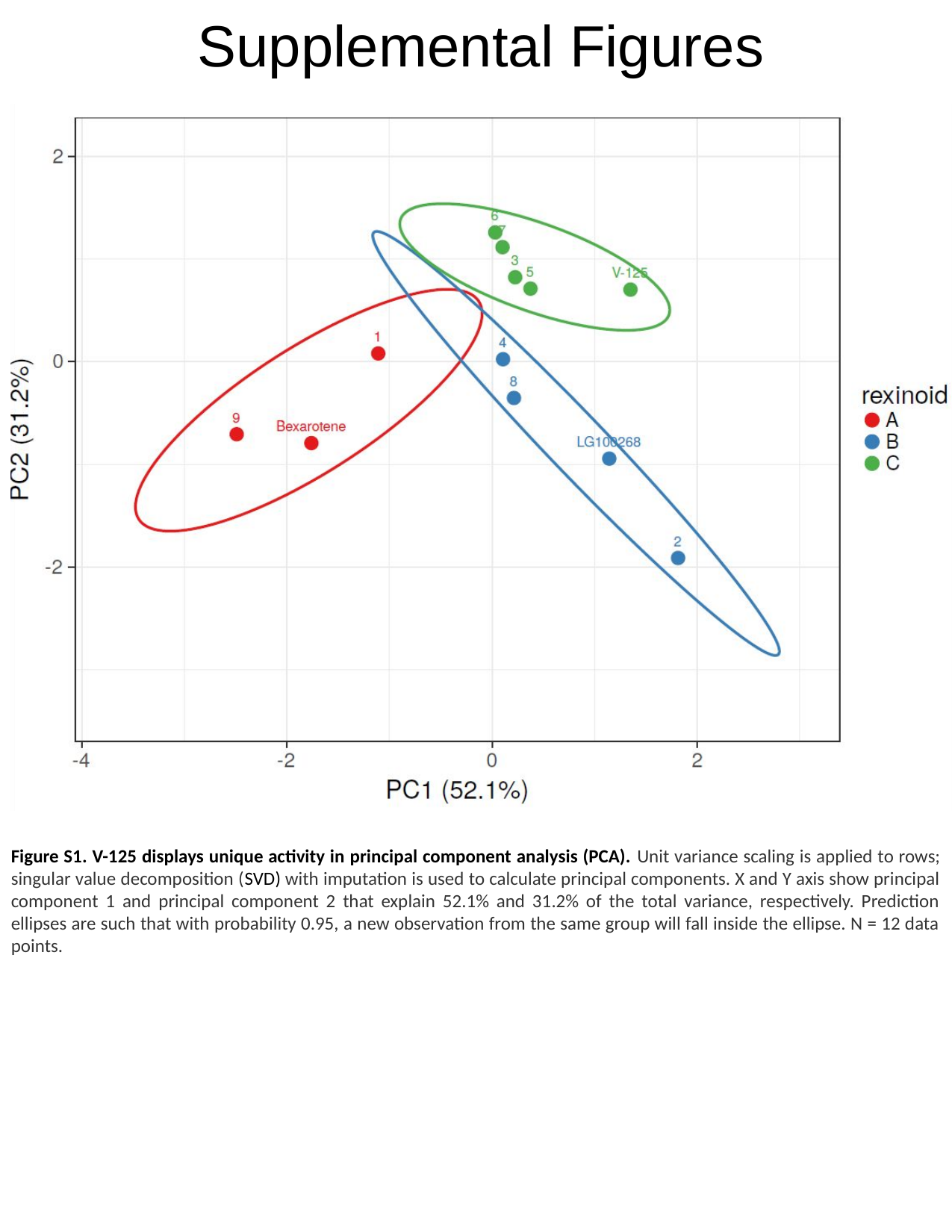

Supplemental Figures
Figure S1. V-125 displays unique activity in principal component analysis (PCA). Unit variance scaling is applied to rows; singular value decomposition (SVD) with imputation is used to calculate principal components. X and Y axis show principal component 1 and principal component 2 that explain 52.1% and 31.2% of the total variance, respectively. Prediction ellipses are such that with probability 0.95, a new observation from the same group will fall inside the ellipse. N = 12 data points.

## Slide 2
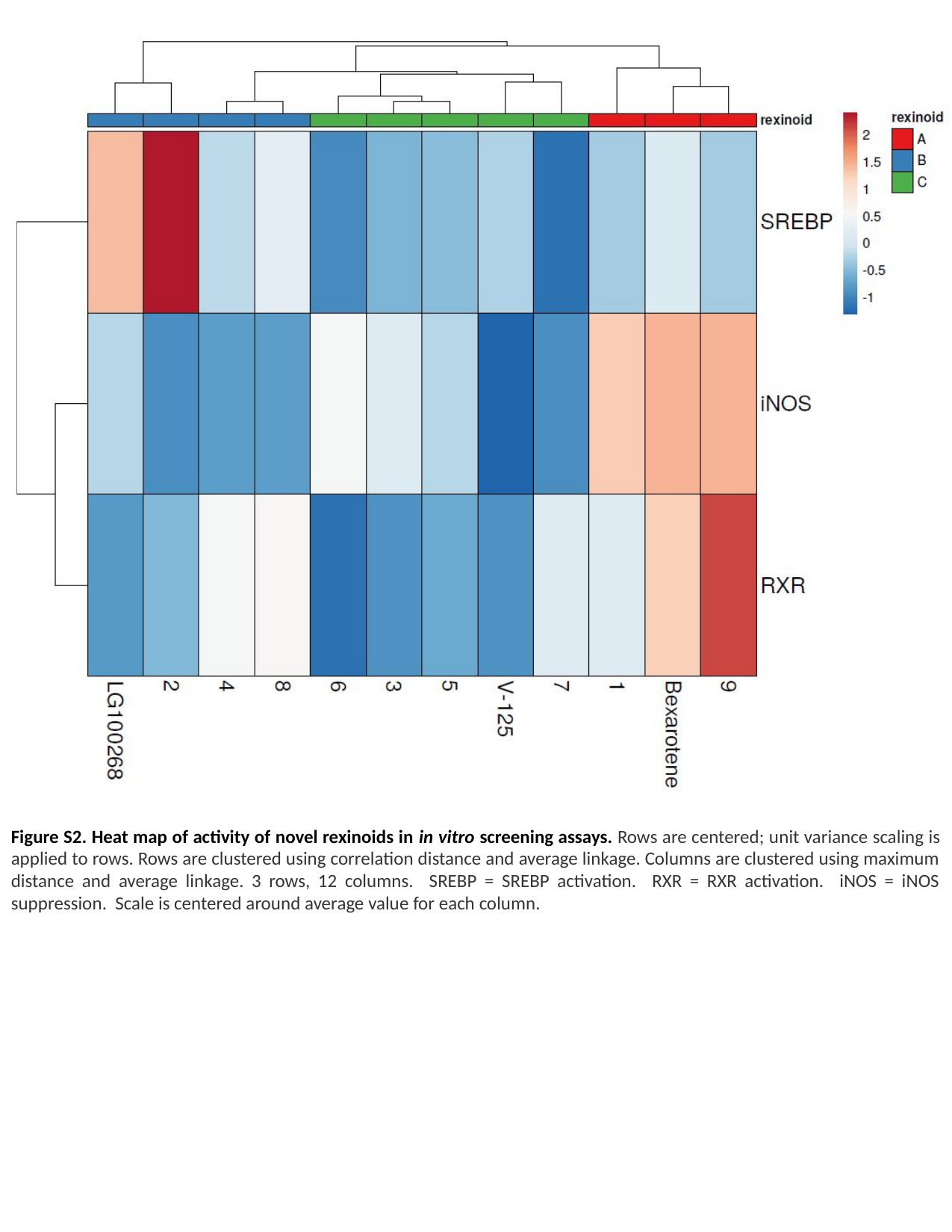

Figure S2. Heat map of activity of novel rexinoids in in vitro screening assays. Rows are centered; unit variance scaling is applied to rows. Rows are clustered using correlation distance and average linkage. Columns are clustered using maximum distance and average linkage. 3 rows, 12 columns. SREBP = SREBP activation. RXR = RXR activation. iNOS = iNOS suppression. Scale is centered around average value for each column.

## Slide 3
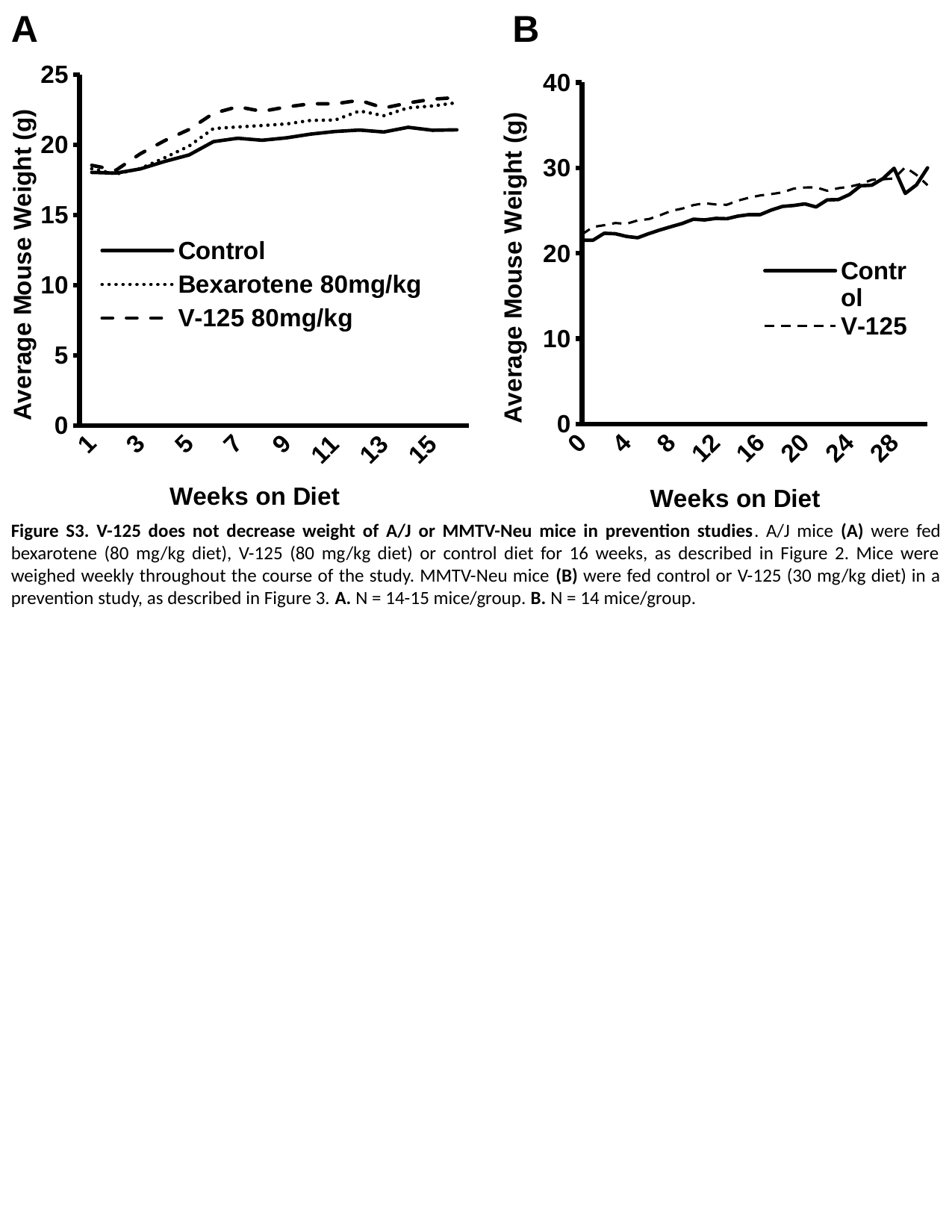

B
A
### Chart
| Category | | |
|---|---|---|
| 0 | 21.51428571428571 | 22.213333333333335 |
| 1 | 21.52857142857142 | 23.08 |
| 2 | 22.342857142857138 | 23.286666666666658 |
| 3 | 22.28571428571428 | 23.54 |
| 4 | 21.97142857142857 | 23.446666666666665 |
| 5 | 21.814285714285713 | 23.846666666666668 |
| 6 | 22.3 | 24.000000000000007 |
| 7 | 22.728571428571428 | 24.446666666666665 |
| 8 | 23.11428571428571 | 24.95333333333333 |
| 9 | 23.485714285714288 | 25.233333333333334 |
| 10 | 23.985714285714284 | 25.640000000000004 |
| 11 | 23.900000000000002 | 25.859999999999996 |
| 12 | 24.078571428571426 | 25.71333333333333 |
| 13 | 24.04285714285714 | 25.673333333333332 |
| 14 | 24.349999999999998 | 26.16 |
| 15 | 24.523076923076925 | 26.513333333333335 |
| 16 | 24.523076923076925 | 26.771428571428572 |
| 17 | 25.066666666666674 | 26.928571428571427 |
| 18 | 25.481818181818184 | 27.12857142857143 |
| 19 | 25.59 | 27.571428571428573 |
| 20 | 25.777777777777775 | 27.700000000000006 |
| 21 | 25.416666666666668 | 27.71428571428571 |
| 22 | 26.25 | 27.316666666666666 |
| 23 | 26.283333333333335 | 27.61666666666666 |
| 24 | 26.879999999999995 | 27.791666666666668 |
| 25 | 27.9 | 28.1 |
| 26 | 27.96666666666667 | 28.590909090909097 |
| 27 | 28.733333333333334 | 28.660000000000004 |
| 28 | 29.95 | 28.749999999999996 |
| 29 | 27.0 | 30.066666666666666 |
| 30 | 28.0 | 29.18 |
| 31 | 30.0 | 27.96666666666667 |
### Chart
| Category | Control | Bexarotene 80mg/kg | V-125 80mg/kg |
|---|---|---|---|Figure S3. V-125 does not decrease weight of A/J or MMTV-Neu mice in prevention studies. A/J mice (A) were fed bexarotene (80 mg/kg diet), V-125 (80 mg/kg diet) or control diet for 16 weeks, as described in Figure 2. Mice were weighed weekly throughout the course of the study. MMTV-Neu mice (B) were fed control or V-125 (30 mg/kg diet) in a prevention study, as described in Figure 3. A. N = 14-15 mice/group. B. N = 14 mice/group.

## Slide 4
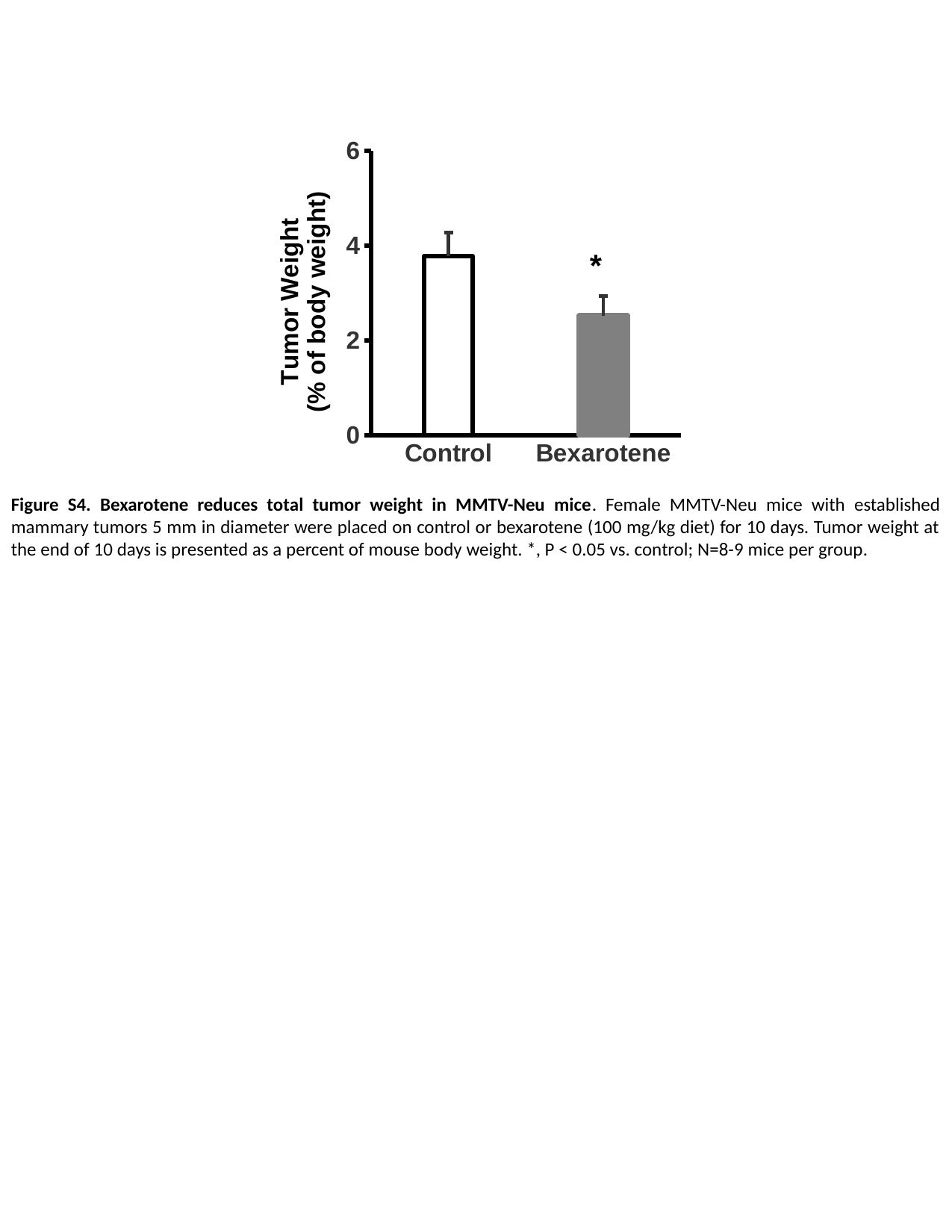

### Chart
| Category | |
|---|---|
| Control | 3.775140928416453 |
| Bexarotene | 2.5248331561464 |*
Figure S4. Bexarotene reduces total tumor weight in MMTV-Neu mice. Female MMTV-Neu mice with established mammary tumors 5 mm in diameter were placed on control or bexarotene (100 mg/kg diet) for 10 days. Tumor weight at the end of 10 days is presented as a percent of mouse body weight. *, P < 0.05 vs. control; N=8-9 mice per group.

## Slide 5
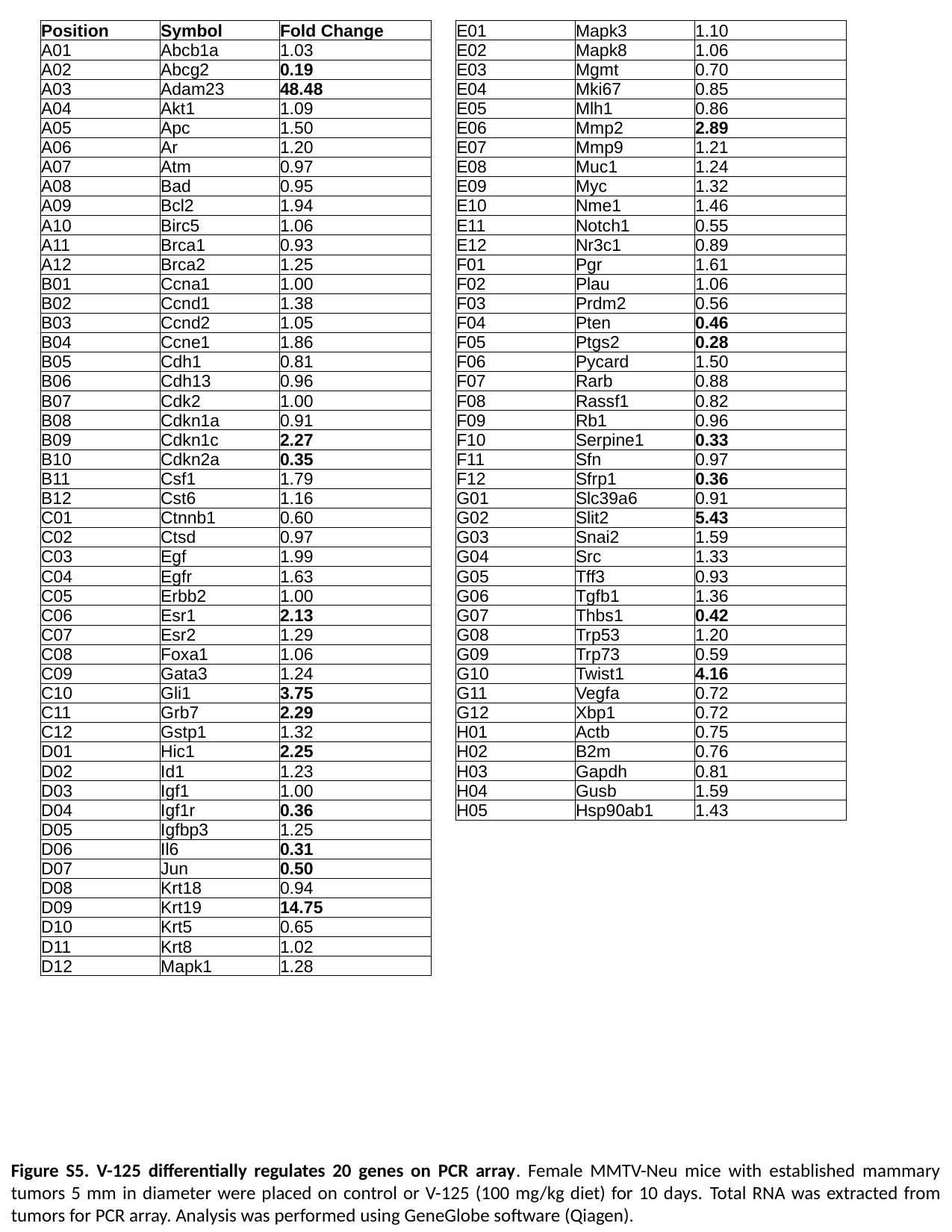

| Position | Symbol | Fold Change |
| --- | --- | --- |
| A01 | Abcb1a | 1.03 |
| A02 | Abcg2 | 0.19 |
| A03 | Adam23 | 48.48 |
| A04 | Akt1 | 1.09 |
| A05 | Apc | 1.50 |
| A06 | Ar | 1.20 |
| A07 | Atm | 0.97 |
| A08 | Bad | 0.95 |
| A09 | Bcl2 | 1.94 |
| A10 | Birc5 | 1.06 |
| A11 | Brca1 | 0.93 |
| A12 | Brca2 | 1.25 |
| B01 | Ccna1 | 1.00 |
| B02 | Ccnd1 | 1.38 |
| B03 | Ccnd2 | 1.05 |
| B04 | Ccne1 | 1.86 |
| B05 | Cdh1 | 0.81 |
| B06 | Cdh13 | 0.96 |
| B07 | Cdk2 | 1.00 |
| B08 | Cdkn1a | 0.91 |
| B09 | Cdkn1c | 2.27 |
| B10 | Cdkn2a | 0.35 |
| B11 | Csf1 | 1.79 |
| B12 | Cst6 | 1.16 |
| C01 | Ctnnb1 | 0.60 |
| C02 | Ctsd | 0.97 |
| C03 | Egf | 1.99 |
| C04 | Egfr | 1.63 |
| C05 | Erbb2 | 1.00 |
| C06 | Esr1 | 2.13 |
| C07 | Esr2 | 1.29 |
| C08 | Foxa1 | 1.06 |
| C09 | Gata3 | 1.24 |
| C10 | Gli1 | 3.75 |
| C11 | Grb7 | 2.29 |
| C12 | Gstp1 | 1.32 |
| D01 | Hic1 | 2.25 |
| D02 | Id1 | 1.23 |
| D03 | Igf1 | 1.00 |
| D04 | Igf1r | 0.36 |
| D05 | Igfbp3 | 1.25 |
| D06 | Il6 | 0.31 |
| D07 | Jun | 0.50 |
| D08 | Krt18 | 0.94 |
| D09 | Krt19 | 14.75 |
| D10 | Krt5 | 0.65 |
| D11 | Krt8 | 1.02 |
| D12 | Mapk1 | 1.28 |
| E01 | Mapk3 | 1.10 |
| --- | --- | --- |
| E02 | Mapk8 | 1.06 |
| E03 | Mgmt | 0.70 |
| E04 | Mki67 | 0.85 |
| E05 | Mlh1 | 0.86 |
| E06 | Mmp2 | 2.89 |
| E07 | Mmp9 | 1.21 |
| E08 | Muc1 | 1.24 |
| E09 | Myc | 1.32 |
| E10 | Nme1 | 1.46 |
| E11 | Notch1 | 0.55 |
| E12 | Nr3c1 | 0.89 |
| F01 | Pgr | 1.61 |
| F02 | Plau | 1.06 |
| F03 | Prdm2 | 0.56 |
| F04 | Pten | 0.46 |
| F05 | Ptgs2 | 0.28 |
| F06 | Pycard | 1.50 |
| F07 | Rarb | 0.88 |
| F08 | Rassf1 | 0.82 |
| F09 | Rb1 | 0.96 |
| F10 | Serpine1 | 0.33 |
| F11 | Sfn | 0.97 |
| F12 | Sfrp1 | 0.36 |
| G01 | Slc39a6 | 0.91 |
| G02 | Slit2 | 5.43 |
| G03 | Snai2 | 1.59 |
| G04 | Src | 1.33 |
| G05 | Tff3 | 0.93 |
| G06 | Tgfb1 | 1.36 |
| G07 | Thbs1 | 0.42 |
| G08 | Trp53 | 1.20 |
| G09 | Trp73 | 0.59 |
| G10 | Twist1 | 4.16 |
| G11 | Vegfa | 0.72 |
| G12 | Xbp1 | 0.72 |
| H01 | Actb | 0.75 |
| H02 | B2m | 0.76 |
| H03 | Gapdh | 0.81 |
| H04 | Gusb | 1.59 |
| H05 | Hsp90ab1 | 1.43 |
Figure S5. V-125 differentially regulates 20 genes on PCR array. Female MMTV-Neu mice with established mammary tumors 5 mm in diameter were placed on control or V-125 (100 mg/kg diet) for 10 days. Total RNA was extracted from tumors for PCR array. Analysis was performed using GeneGlobe software (Qiagen).
